# Physical Culture in the Medical World

**Published:** 1900-09

**Authors:** Mary L. H. Arnold-Snow

**Affiliations:** Late Clinical Assistant in Electro-Therapeutics in N. Y. Post-Graduate Medical School and Hospital


ATLANTA

### Journal-Record of Medicine.

Successor to Atlanta Medical and Surgical Journal, Established 1855, and Southern Medical Record, Established 1870.

VOL. II. SEPTEMBER, 1900. No. 6.

EDITORS { BERNARD WOLFF, M.D., DUNBAR ROY, A.B., M.D. M. B. HUTCHINS, M.D., BUSINESS MANAGER.

PUBLISHED MONTHLY. OFFICE 305 AND 306 FITTEN BUILDING.

### ORIGINAL COMMUNICATIONS.

### PHYSICAL CULTURE IN THE MEDICAL WORLD.

BY MARY L. H. ARNOLD-SNOW, M.D.,

Late Clinical Assistant in Electro-Therapeutics in N. Y. Post-Graduate Medical School and Hospital.

Physical culture in its strictest sense means not the fad gymnastics of the day, not routine exercise for the multitude, but the care or education of man as a whole, the scientific evolution of the human system, its aim being to assist nature or art in relieving, curing, protecting against, or arresting deformity or disease—to develop the wonderful handiwork of God harmoniously.

Athletics have held a place of high recognition for centuries. Did not the ancient Greeks mark religion, victory and honor by periodical games, the Olympian, Delphian, Isthmian and Nemean ? Their gymnasia fostered learning, cleanliness and vigor. Did not Galen say he was "the best physician who was the best teacher of gymnastics"? To Hendicus do we owe the first discovery of the relation between exercise and health, and to Socrates and Hippocrates are we indebted for the early systematizing of medical gymnastics. Later the Romans introduced physical education. Following history, we see the trend of exercise in the thrilling stories relative to the crusades, feudal system and chivalry. As advocates of exercise, we note Luther, Melancthon, Elyot, Dr. Mercurialis, Milton, Montaigne, and in 1893 Locke, who championed medical gymnastics. Pestalozzi directed movements to the joints. Early in German history were education and gymnastics connected, but later the connection was severely strained, one might say, almost severed. At the present time they are more closely united than ever before.

During the last fifty years the science has gone through an era of scintillation. This has been due to various causes. Of what value is it? Is it a pastime, or is it to influence the life of the individual—to appeal to his social nature—and to foster civilization? Wheresoever it be introduced, not until it demonstrates itself of practical importance to man's well-being will it gain a living status in the educational world. View the grand results obtained at Harvard, Yale, Wellesley, etc., where the work has been fully systematized and grounded on a firm medical basis. But such results should not be left to but a few of the leading colleges or gymnasia of our country; they should be of greater scope.

Physical culture is a restorative spoke in the great, nicely balanced wheel of nature. Not until its place is clearly defined will its importance be recognized by the world, and that status will ultimately depend upon the enlightened medical practitioner.

The German, the Swedish, the Delsarte and many other systems have their advocates. Probably the greatest rivalry is between the German and the Swedish systems. The German system has a psycho-physiological base, and aims at general physical culture. It stands for exercise as a whole against sets of muscles. It favors apparatus in its work.

Ling, the founder of the Swedish system, considered a physiological basis necessary, every movement demonstrating its physiological effects. The German system claims to have paid more attention to the nervous system. It "moves with general pedagogy." The Swedish system has "methods and ways of procedure. It begins at definite points and proceeds with order and regularity." The Delsarte is really not a system, but a part of a system, its underlying principles being "relaxation, energizing and deep breathing." Each has its merits, but as a cosmopolitan people, eventually we shall have a truly American system evolved from the best of all.

Rational physical culture should have anthropometry as a base. A complete history should be taken of each case, name, date, age, occupation, weight, family history, previous diseases or accidents, general symptoms, condition of respiratory, circulatory, digestive, genito-urinary, and nervous systems, examination of special senses, heights, girths, breadths, lengths, depths, strengths and deformities. Inspection, palpation, auscultation and percussion are called upon. Great is the importance of a correct diagnosis. Examination of the respiratory system includes many parts. The nose or throat may show the presence of catarrh, the cause of asthma, or even the trace of syphilis. The presence or non-presence of disease should have a direct bearing upon the breathing exercises. Asthma calls for shoulder raising and lowering arm work, having a tendency to elevate the chest. The larynx shows tuberculosis, syphilis, exudations, tumors, stenosis, etc.

Chest examination adds to anthropometrical data. Inspection shows form of thorax, thereby revealing certain causes to the skilled physician. The form of the chest shows the place of need for increased development, but the cause demonstrated by that form shows that the active exercise of certain muscles should be wholly prohibited, while that of others should be mild and under certain regulations. Inspection reveals spinal curvature and bent carriage, for the cure of which suitable exercise should be prescribed. The degree of curvature shows whether the case is suitable for rational gymnastics or the surgeon's art. A thorough examination in respect to palpation shows pain, thoracic movements, fremitus and fluctuation. Pain, bronchial fremitus, or friction fremitus indicates the presence or former presence of inflammatory disease, where rest of the part is essential, and work of its opposite may be necessary. Symmetrical exercise should then be avoided. Although working favorably in health, for a weak chest, head and trunk bending, arm stretching and wand exercise might mean life-long injury to a sufferer. Mensuration, stethometry, pneumatometry and spirometry are essential to assist in demonstrating the present condition of the patient, and later condition after a systematic course of exercise. Percussion may outline viscera, or demonstrates dullness over the lungs, which may mean airless tissue, or exudations, infiltrations, relaxation of lung tissue, etc., which modifies exercise. Auscultation relating to changes in vesicular murmur, râles, etc., demonstrates the prohibition of certain movements. Microscopical examinations and urinary analysis assist in outlining the work of the gymnast. Examination of the heart reveals dislocation, change in size, insufficiency, stenosis, etc., in which cases violent exercise or slight overwork may mean death.

Certain stomach troubles necessitate trunk bending, arm swinging or trunk rotation, although other existing conditions might prohibit or modify this prescription. Anemia would be an indication for head and trunk bending, arm raising, etc., whereas wand exercise would be barred as being too violent and liable to be followed by consequences of overwork. The more accurate the diagnosis, the better the exercises can be regulated to existing needs, and hence the greater the improvement of the patient. We recognize that some colleges require anatomical dissections, etc., in their physical culture course, yet as representatives of Æsculapius we know that such work does not represent a technical medical course. Such work should be referred by the advocate of physical culture to a physician. After the report of the case the physical culture teacher should outline his work. The physician bears the same relation to the physical culture instructor that he does to the pharmacist, or that the oculist does to the optician. When such is recognized will physical education take a step forward.

Some cases are better adapted to exercise than to drug treatment. Other cases of the same name, but of different degree, belong to the physician wholly, while others will eventually yield but to the surgeon. So a technical degree of nicety of discernment must be impartially recognized by the physician making the diagnosis after consultation with the instructor of physical culture, and the case should be relegated to the proper person, be he physician, surgeon or physical culture instructor. Physicians have their domain, the physical culture operators theirs, yet should they be united by "the deepest compassion for human ills, the broadest generosity to human needs, the highest respect for all that is strong and pure and holy in human lives."

Exercise is work done with a physical object, either strength, power or health, and has as a base muscular contraction, which is subordinate to the will. Coordination is essential, and dynamic contractions must be carefully studied with regard to their adaptability. We must ward off objective fatigue due to altered chemical composition—subjective fatigue should be a modifying guide. Exercise causes effort, which has aptly been said to be "a physiological action which consists in the association of a great number of muscles and bones, that they may assist in the same movement, and which, moreover, brings into active association with the muscular work two great functions of the economy: respiration and circulation, a phenomenon associating the great organic functions with the most localized muscular action." More air is taken into the system, making possible greater oxidation with its attendant warmth or glow. Elimination is increased, and dissimilation is accelerated. Cellular activity is increased. Exercise "increases the gains, also the losses of the system, but tends to a more perfect equilibrium of the system in general." It increases the size of the chest, strengthens the muscles, increases the capacity of the lungs. It flushes out the great channels of the body. Surplus fat serves as fuel, the muscle tissue, receiving increased nutrition, changes in size and shape. It becomes firm and reveals its curves or beauty. But if overworked a diminution in size occurs, or even rupture.

It has been ably demonstrated by Bernard Roth, F.R.C.S., that exercise has a marked effect on certain spinal curvatures. It lifts the drooping shoulders of the child, it trains the wayward and awkward carriage of the body, so often later calling for surgical measures. Passive, resistive or active flexion, extension, pronation, supination, abduction, adduction and circumduction have welldefined fields that yield marked results under the guidance of a skilled physical culture operator, that naturally aids the surgeon or physician in the treatment following fractures, for weak muscles, occupation neuroses, paralysis, lack of development, nutritive disorders, anemia, general debility, inactivity of organs, plethora, asthma, constipation, etc. In some cases it is able to work alone, in others it simply assists. This is where discrimination must be made, and neither the physical culture teacher nor the physician or surgeon should hesitate to honestly acknowledge when a case is beyond his specialty.

Another side to be considered by the physician is that of mental fatigue. In this age of civilization when "cram" is the watchword, many are the children who suffer, often silently, from overwork. In such cases tonics, fresh air, and special drug treatment have a place, but properly adjusted exercise helps to establish a body equilibrium. The child may take exercise at school; but, pray, do not blame the overworked and usually underpaid teacher, for he does the best he can under existing circumstances. In classes of thirty or even fifty can individual attention be given? The exercises must be class work, they must call forth the energies of the mind, the pupil's close and fixed attention, judgment and comparison, in order that any work may be done, yet at the same time additional work is being put upon the brain. Economy of mental energy must be recognized by not only the physician and the instructor of physical culture, but by the masses.

Properly applied gymnastics also act as a preventive of certain bodily tendencies, and are advisable for healthy individuals. But we shall make no further note of this side of the question.

Physicians, men of broad culture, men of highest intellectuality, those who "come closest to the mystery of life and the mystery of death, who read the naked heart when it is too weak or too sorrowful to hide its nakedness," add one more panacea to human ills. Consider the subject of physical culture, analyze its merits and demerits; give it an impartial trial in connection with medical, surgical, hot-air, or electrical treatment. You are progressive, thoughtful, keen, and recognizing the importance of a healthy mind, and knowing that "mens sana in corpore sano," broaden your field and power to give such, and ascend one more round in the ladder of medical advancement for the benefit of that countless multitude of humanity who honor and respect, yea, almost worship, "Our Doctor," who brings sunshine, health, and consequent happiness to serve in life's battle.

